# Effect of Irradiated and Non-Irradiated Waste PET Based Cementitious Grouts on Flexural Strength of Semi-Flexible Pavement

**DOI:** 10.3390/ma12244133

**Published:** 2019-12-10

**Authors:** Muhammad Imran Khan, Huang Yong Huat, Mohammad Haziq bin Muhamad Dun, Muslich Hartadi Sutanto, Ehsan Nikbakht Jarghouyeh, Salah E. Zoorob

**Affiliations:** 1 Department of Civil & Environmental Engineering, Universiti Teknologi PETRONAS, Seri Iskandar 32610, Malaysia; yonghuat_20113@utp.edu.my (H.Y.H.); mhaziq_20742@utp.edu.my (M.H.b.M.D.); muslich.sutanto@utp.edu.my (M.H.S.); ehsan.nikbakht@utp.edu.my (E.N.J.); 2 Construction and Building Materials Program, Kuwait Institute for Scientific Research, Safat 13109, Kuwait; szoorob@kisr.edu.kw

**Keywords:** polyethylene terephthalate, flexural strength, irradiation, geopolymer, flowability

## Abstract

In this study the effect of irradiated and non-irradiated waste polyethylene terephthalate (PET) as replacement of cement and fly-ash in ordinary Portland cement (OPC) and geopolymeric cement (GPC) based cementitious grouts on flexural strength of semi-flexible pavement specimens were evaluated. The porous asphalt gradation was selected based on Malaysian specifications for semi-flexible pavements with a target of 30% air voids. The cement content in the OPC grouts and the fly-ash content in the GPC based grouts were partially replaced with 1.25% PET (using both irradiated and non-irradiated PET). Beam specimens were prepared and tested for flexural strength properties using center point loading configuration. The grouts modified with recycled waste plastic (PET) showed approximately the same results as obtained from the control specimens. Although the replacement amount was low (1.25% by weight of cement), nonetheless, significant impact on reducing CO_2_ emissions is expected when preparing grouts for mass construction of semi-flexible pavement surfaces. Similarly, effective recycling of waste plastics in road construction and replacing OPC with plastic and geopolymers will have a positive effect on the environment and will furthermore contribute to sustainable pavement construction.

## 1. Introduction

The aim of pavement construction is to sustain traffic loads and environmental stresses during its design life. Ride quality, passenger safety, life cycle costing, engineering performance, durability and resilience are some of the key criteria that have to be considered during a comprehensive pavement design process. Traditionally, there has been two main choices of road construction; flexible and rigid pavements. Flexible pavements are constructed from bituminous materials and load distribution to lower pavement layers is a function of aggregate–bitumen adhesion, binder rheology, aggregate interlocking and particle friction. On the other hand, rigid pavements are constructed from cement concrete composites with or without reinforcement that have adequate flexural strength to dissipate and transfer the traffic loading to the subgrade through slab action. However, conventional flexible pavements are more susceptible to rutting and fatigue distresses when subjected to heavy traffic loading and adverse weather conditions in part due to their low durability. Rigid pavements have high construction costs and typically slow concrete setting times, as well as rough ride quality due to joints. Hence, due to some constraints and demerits of both flexible and rigid pavements, a hybrid type of pavement composite, named semi-flexible pavement, has been gaining popularity in recent years due to its advantages over both flexible and rigid pavements [1]. Semi-flexible pavements are comprised of a porous asphalt skeleton with 20–35% voids, over which a highly flowable cementitious grout is spread and allowed to infiltrate [2,3]. Researchers have identified several factors that affect performance of cementitious grouts and semi-flexible pavement surfaces and these are summarized in the following sections.

### 1.1. Water–Cement Ratio

Water–cement ratio (w/c ratio) plays a significant role in the fluidity and strength properties of grouts. For semi-flexible pavement, the grouts are required to be highly flowable with desired strength. Various ranges of w/c ratios (from 0.35 to 0.63) have been optimized by researchers to achieve the target fluidity and strength of different grouts for semi-flexible surfaces [4,5,6,7,8,9,10,11]. 

### 1.2. Admixtures, By-Products and Other Supplementary Materials

Researchers have also been investigating ways to replace ordinary Portland cement (OPC) partially or fully with wastes or by-products and other cementing materials, in order to minimize the use of cement in the construction industry, which may help to reduce CO_2_ emissions. Fly ash, ground granulated blast furnace slag, mild glass, Panasqueira waste mud, geopolymeric grouts and crosslinked polymer resin have been used as a cement replacement in the grouts to investigate the physical and mechanical performance of cementitious grouts for semi-flexible pavements. All these additives exhibit positive impacts on fluidity and strength properties of cementitious grouts [5,12,13,14,15]. The effect of rubberized based cement grouts has also shown advantages in terms of reduced shrinkage, higher freeze thaw resistance, reduced tyre-pavement noise pollution, but unfortunately this is accompanied by a reduction in mechanical properties [16]. In another study, high performance cement was produced by using different percentages of polycarboxylate superplasticizer, UAE expensive agent (U-type expansive agent for concrete admixture) and high performance air-entrained agent, and the pavement performed better in terms of moisture susceptibility, rutting resistance and mechanical properties [17]. Furthermore, a study was conducted to evaluate the cracking resistance of semi-flexible pavement incorporating modified asphalt with Styrene–Butadiene–Styrene, Modified Agent-10 (MA agent) and fibers. The results indicated that the addition of these modifiers reduces the strength, but improves the fatigue (cracking) resistance of semi-flexible pavements [18]. In another study it was concluded that latex modified cement grout for grouted macadam showed significant improvement against rutting, moisture damage, low temperature performance and improved flexural strength, while only slightly compromising on fluidity [19]. 

### 1.3. Voids in Porous Asphalt Mixtures

Voids ratio or porosity have a significant effect on the final properties of semi-flexible specimens. If porosity is too low, voids may not be interconnected, and grouts will not be able to penetrate fully. On the other hand, if porosity is too high, the porous asphalt skeleton will require an excessive quantity of cementitious grout and the final product will behave as a rigid composite. Researchers have come up with a range voids ratio (from 20% to 35%) to achieve the optimum balance of strength properties of semi-flexible specimens [20,21].

### 1.4. Bitumen Type and Aggregate Gradation

The physical and performance properties of semi-flexible specimens also depend on the type of bitumen and choice of aggregate gradation. Polymer modified bitumen could be a better choice to improve the stiffness and strength of semi-flexible specimens. Similarly, improved high and low temperature performance grades using polymer modified bitumen would be a better choice to counter the effect of extremes in environmental temperatures [20]. Similarly, aggregate gradation for porous asphalt skeleton shall be properly designed to achieve the target high porosity (20–35%). Gradation has an important impact on resilient modulus and strength properties of semi-flexible specimens. Gradation with increased percentage of fine aggregates leads to less porous structure and hence grouts are not capable of full depth penetration, which causes reduction in both strength and abrasion resistance of semi-flexible specimens [22,23].

Due to rapid urbanization/industrialization and population growth, the production of municipal wastes has been increasing significantly in Malaysia. The primary mode of disposal of these wastes is in dumpsites or landfills in Malaysia. A survey conducted by the National Solid Waste Management Department shows that plastics, at 13.2% by mass of total municipal waste, is the second highest waste type after organic waste (44.5%) generated from households [24]. Polyethylene terephthalate (PET) is one of the most widely used plastic types mostly used for soft-drinks containers due to its durable, tough nature. The majority of the plastics that are collected as recycling waste from households are plastic bottles and 50–60% of these bottles are manufactured from PET [25]. 

The aim of this study was to determine the best combination of w/c ratio and dosage of superplasticizer to fulfill the flowability requirement of grouts, with optimized compressive strength. Concurrently, in view of environmental issues and the low recycling rate of PET in Malaysia, the effect of irradiated and non-irradiated waste polyethylene terephthalate (PET) based cementitious grouts on flexural strength of semi-flexible specimens were evaluated in the current study. The purpose of exposing PET to gamma irradiation was to explore an effective way of recycling PET in cementitious grouts. Irradiation of PET is an innovative way of recovering back the strength which has been lost due to using normal plastic. The irradiation is was conducted by exposing PET to 100 kGy of gamma rays and this helps to improve the cross-scission and cross-linking behavior of the PET polymer. Utilizing waste PET in road construction will help the environment by recycling waste plastics instead of dumping in landfills or floating in rivers. Similarly, replacing cement with wastes and by-products (such as fly ash) will also help in minimizing CO_2_ emissions.

## 2. Materials and Methods

### 2.1. Material Selection

**Figure 1:**
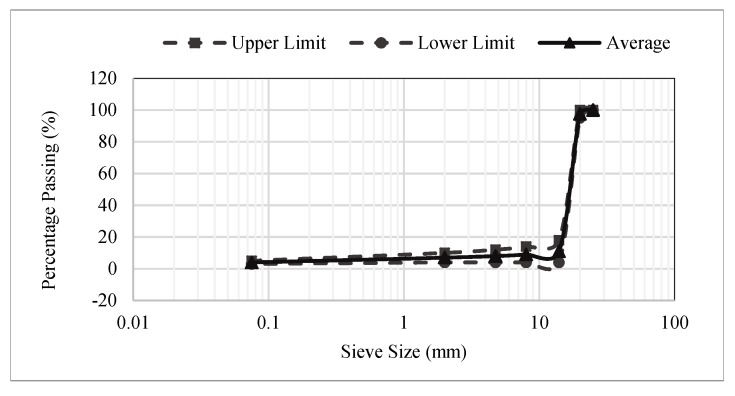
Porous aggregate gradation as per REAM specifications [26].

Local limestone aggregates, ordinary Portland cement and bitumen (Penetration grade 60/70) were used in this study. The mix composition and aggregate gradation were selected according to Road Engineering Association Malaysia (REAM) specifications [26] as shown in Table 1 and Figure 1. Waste polyethylene terephthalate (PET) was collected from the Enhance Plastic Factory, Malaysia and was irradiated by 100 kGy at the Malaysian Nuclear Agency, Kuala Lumpur, Malaysia. The PET was further ground into powder (size < 75 µm) for the purpose of partially replacing cement in cementitious grouts. Geopolymer cement (GPC) was prepared in laboratory settings and the compositions are shown in Table 1. In the current study, cement and fly ash was partially replaced by 1.25% PET in OPC and GPC based grouts respectively. This percentage was selected based on a study conducted by MIT [27]. Similarly, chemical and elemental composition of OPC and fly ash are given in Table 2. The percentage contribution of Al_2_O_3_, SiO_2_ and Fe_2_O_3_ is less than 70% and hence this fly ash fulfills the requirement of high calcium fly ash in accordance with ASTM 618-10. Fly ash was procured from Manjung Power Plant, Sitiawan, Perak, Malaysia. Polycarboxylate ether type superplasticizer was used in this study and was obtained from BASF Sdn Bhd, Shah Alam, Malaysia. 

### 2.2. Preparation of Grouts

Initially five combinations of grouts were prepared by varying the w/c ratio from 0.25 to 0.45. Similarly, 1% superplasticizer was also used to achieve high flowability in grouts. A mechanical mixer was used for mixing the required amount of materials in accordance to the ASTM C305-14 [28]. In preparing the OPC based grouts, the cement and 1.25% PET was first mixed in dry condition and then the required amount of water and superplasticizer was added and further mixed as per ASTM C305-14. Similarly, in preparation of the GPC grouts, the composition of geopolymer was selected from a previous study that was conducted in the laboratory [29]. Sodium hydroxide (NaOH) with 99% purity and Sodium silicate (Na_2_SiO_3_) as an alkaline activator were used. The mixing ratios are given in Table 1 for producing geopolymeric grouts. Initially, the alkaline solution was added to the fly ash and mixed in a Hobart mechanical mixer for 30 s. Afterward all chemicals were added and further mixed for 4 min on fast speed.

Flow test was conducted on all grouts using Malaysian Flow cone to measure the flow-out time of grouts [26,30]. Furthermore, cubes (50 × 50 × 50 mm^3^) were also prepared to test the grouts for compression test at 1-day, 7-days and 28-days curing using ELE Universal Testing Machine with capacity of 3000 kN was used at a loading rate of 0.90 kN/s [31].

### 2.3. Mix Design of Porous Asphalt Skeleton

Aggregates and bitumen were heated and mixed in a laboratory mixer according to ASTM D6926 standards [32]. The mixture was poured into Marshall molds and compacted with 50 blows each end. These number of blows are required for porous specimens, as extra blows causes crushing of aggregates. The specimens with various bitumen contents (in the range 3–4%) were subjected to Marshall stability, density and voids analysis to determine the optimum bitumen content [33]. Bitumen content of 3.2% by weight of total mix was determined as being optimum.

### 2.4. Preparation of Semi-Flexible Specimens

**Figure 2:**
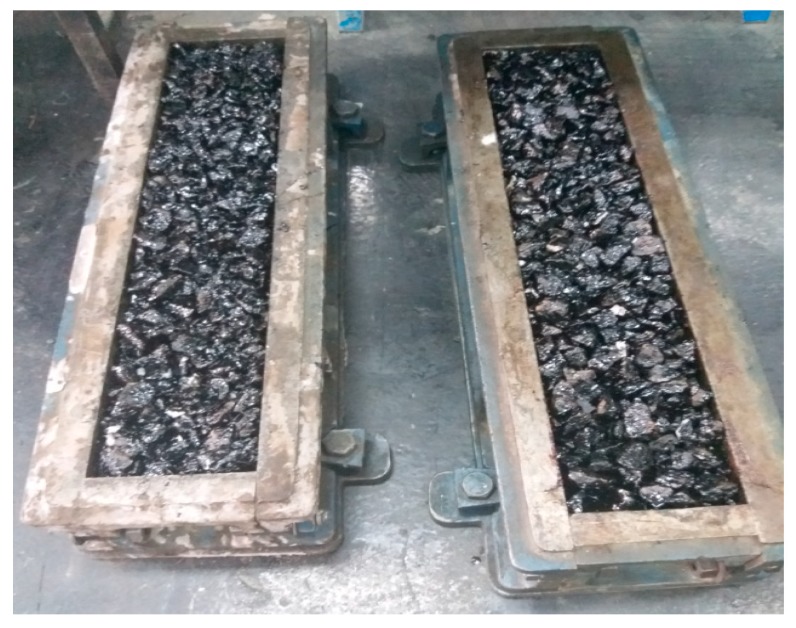
Beam samples of porous asphalt mixtures for flexural test.

After determining the optimum bitumen content, beam specimens of size 150 × 150 mm^2^ and 500 mm length were prepared from porous asphalt mixtures as shown in Figure 2.

The target voids ratio of 30% was achieved to ensure that the voids were interconnected to allow grouts to infiltrate. After one day, the cementitious grouts as shown in Table 2 were prepared. Enough quantity of each grout was spread on the surface of the beam specimens and allowed to penetrate due to gravity, followed by vibration. The semi-flexible beam specimens were demolded after 24 h and kept for 1-day and 28-days in open air for curing purposes.

**Figure 3:**
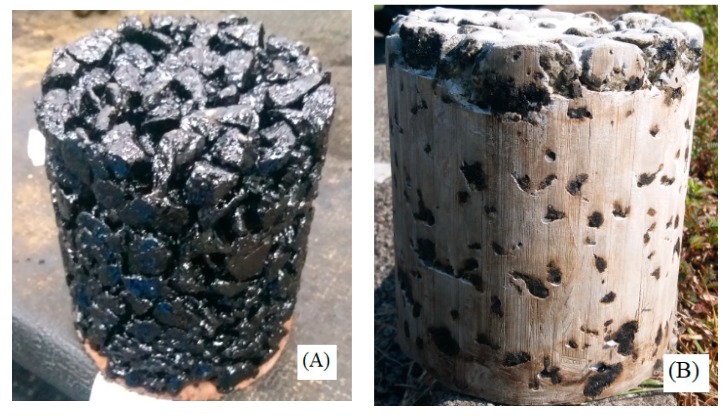

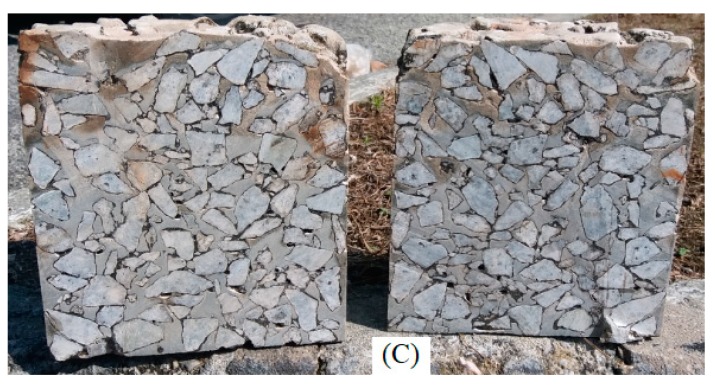
Gyratory compacted specimen (**A**) porous asphalt specimen (**B**) specimen after grout infiltration (**C**) sliced semi-flexible specimen.

Similarly, a Gyratory sample of 6” diameter was prepared from porous asphalt mixture with target voids of 30% and cement grout of same composition was poured on the surface and allowed to infiltrate. The Gyratory specimen was then sliced into two pieces as shown in Figure 3. The purpose was to ensure that the proposed grout was fully penetrated throughout the specimen depth.

It is clear from the sliced specimen (Figure 3C) that the proposed grout had fully penetrated the depth of the specimen, which was an indication of interconnected voids and proper mix design of grout.

### 2.5. Flexural Test of Beam Specimen

**Figure 4:**
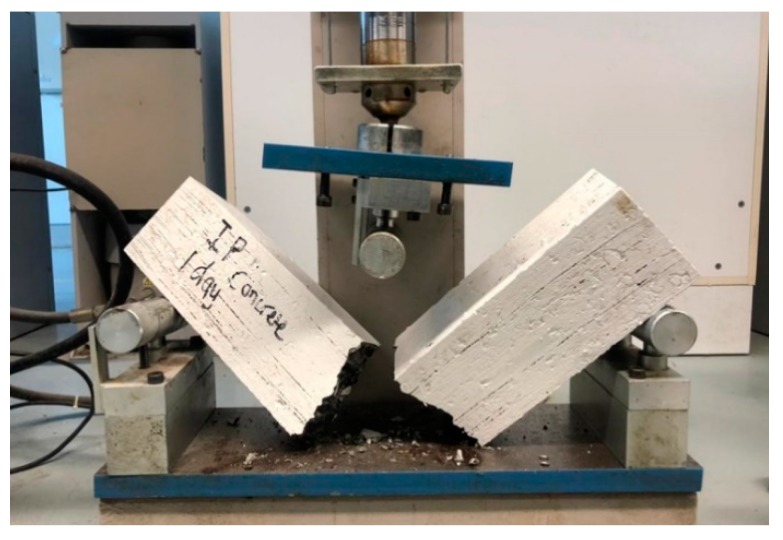
Experimental test to measure flexural strength of semi-flexible beam.

Three-point bending system (or center-point loading) was used to investigate the flexural strength of semi-flexible beam specimens. The resistance to bending failure of unreinforced beams are measured using this test method. The flexural strength is expressed as Modulus of Rupture (MR) and is determined according to ASTM C293 and C78 test methods [34]. The semi-flexible beam specimens were subjected to center load until rupture (see Figure 4). All the mixtures were tested in triplicate and the average results were used for final analysis. 

The load was measured at the failure of samples and the flexural strength is measured from the equation below [34]:

$$\sigma =\frac{3FL}{2bd^{2}}$$

where *σ* = flexural strength (MPa), *F* = maximum load at failure (N), *L* = span length (mm), *b* = width of samples (mm) and *d* = thickness of sample (mm).

## 3. Results and Discussions

### 3.1. Flow and Compressive Strength of Initial Grouts

**Figure 5:**
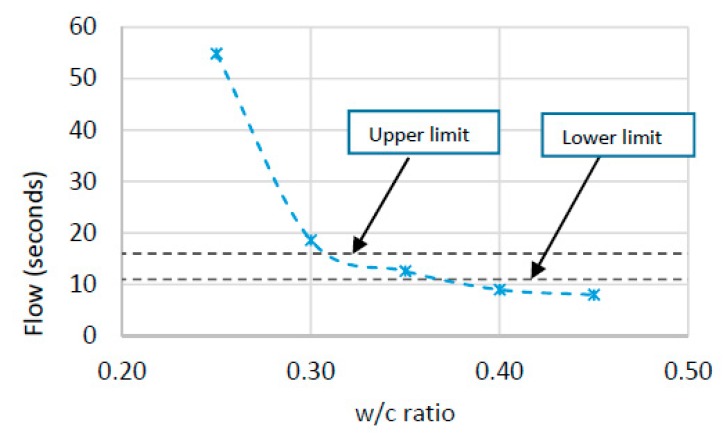
Flow of cementitious grout at different w/c ratios.

**Figure 6:**
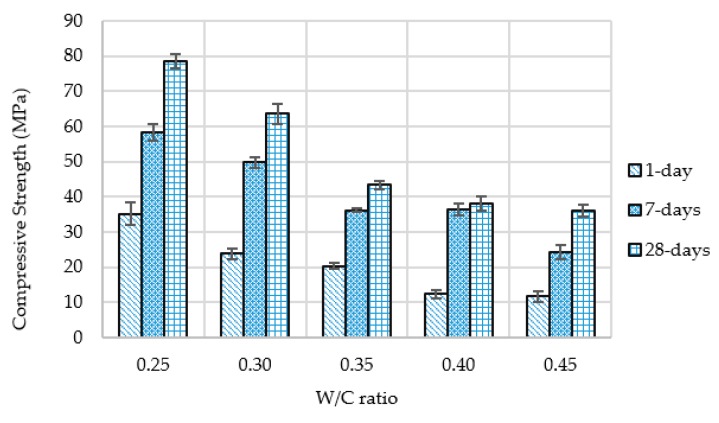
Compressive strength (1-day, 7-days and 28-days) at different w/c ratios.

Based on the results from the flow and compression tests (1-day, 7-days and 28-days) w/c ratio was selected to produce final grouts for semi-flexible specimens. Figure 5 and Figure 6 show the flow and compression test results of grouts respectively. 

It can be seen from Figure 5 that there is a significant drop in flow-out time with an increase in w/c ratio. Initially, this drop is substantial when the w/c ratio increases from 0.20 to 0.30 and then a gradual decrease is witnessed with the addition of more water. According to Malaysian specifications, the flow-out time for grout should be 11 s to 16 s and based on these limitations, only the grout with w/c ratio of 0.35 fulfills the requirement of flowability as shown in Figure 5. Furthermore, by increasing the w/c ratio, the cementitious grouts depicted drop in compressive strength at all curing ages, as shown in Figure 6. This is due to the creation of more pores with increased w/c ratio that causes reduction in strength properties. However, an increase in compressive strength is witnessed with increasing curing time for all grouts. 

### 3.2. Flexural Strength of Semi-Flexible Specimens

#### 3.2.1. OPC Based Semi-Flexible Beams

**Figure 7:**
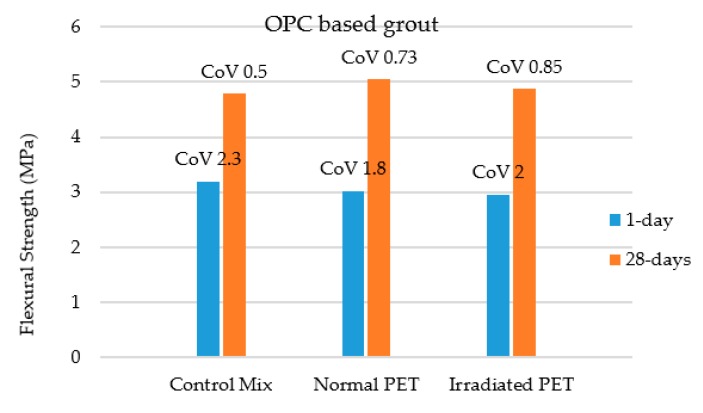
Flexural strength for ordinary Portland cement (OPC) based semi-flexible beams at 1 and 28-days curing.

Flexural strength of irradiated and non-irradiated PET based grouts for semi-flexible specimens were investigated by center point loading system. It is clear from the results shown in Figure 7 that the flexural strength of semi-flexible specimens containing 1.25% PET (normal and irradiated) based grouts showed similar results of flexural strength as compared to control specimens at 1-day curing. The cement in the grout was replaced by 1.25% normal as well as irradiated PET. Similar results of flexural strength of semi-flexible specimens were witnessed at 28-days curing for control and PET based grouts as shown in Figure 7.

However, the percentage of strength gained at 28-days for PET based grouts was more than 60%, while for control grouts it was 50% of early 1-day strength as shown in Figure 7. 

Statistical analysis using t-test with significance level (α) of 0.05 and coefficient of variance (CoV) was conducted to assess the variability of results and the effect of added PET on flexural strength. The statistical analysis on OPC based grouts show that adding 1.25% PET (irradiated and normal) does not cause reduction in flexural strength, thus having high potential for partially replacing Portland cement with PET. It can be seen from the results of t-test as shown in Table 3 that the *p* value is greater than α and hence there is similar flexural strength of all mixtures. Further study is recommended to incorporate more PET for higher impact on sustainability.

#### 3.2.2. GPC Based Semi-Flexible Beams

**Figure 8:**
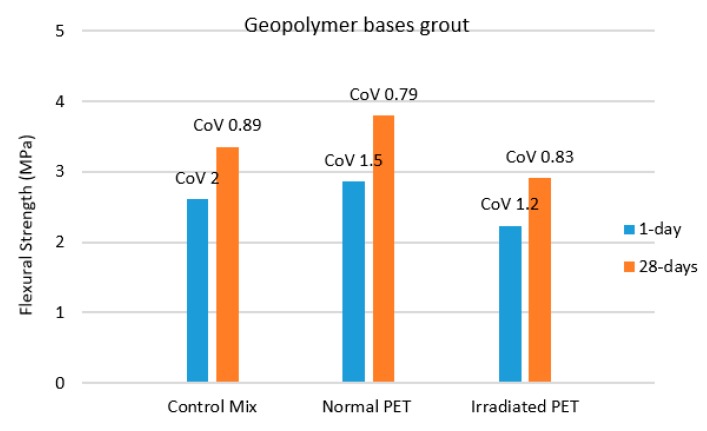
Flexural strength for geopolymeric cement (GPC) based semi-flexible beams at 1 and 28-days curing.

The flexural strength of geopolymer cementitious grouts based semi-flexible beams were also investigated by center point loading system. In geopolymer grouts, the fly ash was replaced by 1.25% of PET (normal and irradiated plastics) and the effect on flexural strength is shown in Figure 8. The results show that there is a slight increase in flexural strength of semi-flexible specimens when fly ash in geopolymer grout is partially replaced by normal PET. However, reduction in flexural strength was observed when irradiated PET was used as partial replacement of fly ash in GPC grouts.

The statistical analysis on GPC based grouts show that adding 1.25% normal PET significantly increased flexural strength. Although it has higher final strength, it is recognized that GPC typically was slower in gaining the strength compared to that of OPC. It seems that the addition of 1.25 untreated PET had a positive impact in supplementing the slower strength gain of GPC, thus showing significant improvement in flexural strength compared to that of OPC based grout that did not show any difference. It is interesting to note that adding 1.25% irradiated PET on GPC grouts reduced the flexural strength. Similarly, results obtained from the t-test as shown in Table 4 demonstrates that the *p* value is less than α and hence, significant variation in flexural strength exists. This might be caused by the physicochemical impact of irradiated PET on GPC grouts, which should be investigated further using morphological testing in future research.

## 4. Conclusions

Irradiated polyethylene terephthalate (PET) was used to partially replace cement in OPC based grouts and fly-ash in GPC based grouts in the formulation of composites for use in semi-flexible pavement applications. Center point loading system was conducted to evaluate the flexural strength of semi-flexible specimens. The following main conclusions were drawn:

1. Addition of superplasticizer (1% by weight of cement) increases the fluidity of grouts, which allows cementitious grouts to fully penetrate through the porous asphalt skeleton.
2. Replacement of cement with waste PET in cementitious grout for semi-flexible specimens showed similar flexural strength properties at 1-day and 28-days curing in comparison to the control mixtures.
3. The strength gained by PET based grouts at 28-days curing is more than 60% higher than 1-day curing, while the increase was 50% for the control specimens. The results implied that strength gained in PET based grouts were more than that of control mix.
4. Normal PET based GPC grouts showed 9–10% increase in flexural strength of semi-flexible specimens as compared to control samples.
5. There is reduction in flexural strength of irradiated PET based GPC grouts by 22–23% and 13–14% as compared to normal PET and control samples respectively.
6. The utilization of waste plastic and fly-ash as a cement replacement can have a positive impact on the environment in terms of recycling plastic wastes and industry by-products in pavement construction.

## Figures and Tables

**Table 1:** Mix composition for porous asphalt and cementitious grouts for semi-flexible specimens.

Mix Composition for Porous Asphalt Structure [26]		Compositions of Grouts		
Composition	Weight (%)	OPC based Grouts		GPC based Grouts
Bitumen	3.0–4.0%	w/c ratio	0.35	Na_2_SiO_3_:NaOH (3:1)
Filler (OPC)	4.0%	Superplasticizer	1% by weight of OPC	NaOH:H_2_O (32:100)
Aggregate	92–93%			
Voids content	20–30%	PET (Irradiated and Non-Irradiated)	1.25% by weight of OPC	Superplasticizer (1% by Fly-Ash)

**Table 2:** Chemical composition of cement and fly ash.

Material/	SiO_2_	Al_2_O_3_	Fe_2_O_3_	CaO	MgO	K_2_O	SO_3_	TiO_2_	P_2_O_5_	Na_2_O	Others
Cement	22.65	4.63	2.34	61.72	4.23	1.14	2.24	0.20	0.12	0.11	0.62
Fly Ash	36.4	13.72	18.24	19	3.26	2.2	2.5	1.45	1.2	1.73	0.3

**Table 3:** Statistical analysis for verification of variable’s similarity in OPC based mixtures.

**1-day Curing**			
	*Control Mix*	*Normal PET*	*Irradiated PET*
Mean	3.19	3.01	2.95
Variance	2.28	1.89	2
P(T ≤ t) one-tail	0.4	0.06	0.37
t Critical one-tail	2.91	2.91	2.91
P(T ≤ t) two-tail	0.81	0.12	0.75
t Critical two-tail	4.3	4.3	4.3
**28-days Curing**			
	*Control Mix*	*Normal PET*	*Irradiated PET*
Mean	4.78	5.04	4.86
Variance	0.47	0.73	0.84
P(T ≤ t) one-tail	0.38	0.42	0.46
t Critical one-tail	2.91	2.91	2.91
P(T ≤ t) two-tail	0.76	0.84	0.93
t Critical two-tail	4.3	4.3	4.3

**Table 4:** Statistical analysis for verification of variable similarity in GPC based mixtures.

**1-day Curing**			
	Control Mix	Normal PET	Irradiated PET
Mean	2.61	2.86	2.23
Variance	2	1.5	1.2
P(T ≤ t) one-tail	0.0006	0.001	0.005
t Critical one-tail	2.91	2.91	2.91
P(T ≤ t) two-tail	0.0012	0.003	0.011
t Critical two-tail	4.3	4.3	4.3
**28-days Curing**			
	Control Mix	Normal PET	Irradiated PET
Mean	3.35	3.79	2.91
Variance	0.89	0.79	0.83
P(T ≤ t) one-tail	0.00034	0.00005	0.0003
t Critical one-tail	2.91	2.91	2.91
P(T ≤ t) two-tail	0.0002	0.0001	0.0006
t Critical two-tail	4.3	4.3	4.3
